# Photon‐counting computed tomography for stopping power ratio prediction in proton therapy

**DOI:** 10.1002/mp.70146

**Published:** 2025-11-18

**Authors:** Sophie Huijskens, Marcel van Straten, Patricia Cambraia Lopes, Linda Rossi, Patrick Wohlfahrt, Mischa Hoogeman

**Affiliations:** ^1^ Erasmus MC Cancer Institute, University Medical Center Rotterdam Department of Radiotherapy Rotterdam the Netherlands; ^2^ HollandPTC Delft the Netherlands; ^3^ Erasmus MC, University Medical Center Rotterdam Department of Radiology & Nuclear Medicine Rotterdam the Netherlands; ^4^ Siemens Healthineers Varian, Cancer Therapy Imaging Forchheim Germany

**Keywords:** photon counting computed tomography, stopping power ratio

## Abstract

**Background:**

A substantial source of error in proton therapy is predicting the stopping power ratio (SPR) of tissues from computed tomography (CT). Due to its systematic nature, it is crucial to minimize this error as much as possible. Photon‐counting CT (PCCT) scanners, which utilize semiconductor detectors to resolve the energy of X‐rays, can extract more information per voxel compared to conventional CT scanners, offering potential improvements in material characterization and higher accuracy in proton range estimation.

**Purpose:**

This study explores the potential of PCCT for improving SPR predictions compared to conventional single‐energy CT (SECT) and dual‐energy CT (DECT).

**Methods:**

SECT, DECT and PCCT scans of the CIRS SPR/Density phantom with tissue‐equivalent inserts were acquired in body and head configurations using comparable scan and quantitative reconstruction settings. The SPR of each insert was predicted from SECT (SPR_SECT_) using a clinical Hounsfield look‐up table (HLUT) and directly derived from DECT (SPR_DECT_) and PCCT (SPR_PCCT_) scans using spectral information (DirectSPR application, provided by Siemens Healthineers). Results were compared against measurements (SPR_Measured_), obtained via a multi‐layer ionization chamber, and differences relative to water (∆SPR) were calculated. Mean absolute errors (MAE) over the inserts were calculated for each imaging modality in both body and head configurations and per tissue subgroup. As a proof‐of‐concept, proton plans for a lung and neurological case were created to assess the impact on dose distribution using the SECT‐HLUT approach (with 3% range uncertainty) or DirectSPR from PCCT (with 2% range uncertainty).

**Results:**

PCCT predicted SPR within 1.0% agreement with SPR_Measured_, except for the insert “100% gland” (1.4%). The largest ∆SPR observed across all inserts and imaging modalities were found in lung and adipose inserts. For the soft tissue and bone inserts, estimated SPRs generally agreed well with SPR_Measured_ (∆SPR ≤1.0%), except for the inserts soft tissue grey and cortical bone (both ∆SPR_SECT,head_ = 1.3%). Among the three imaging modalities, the overall mean absolute error (MAE) was lowest for PCCT by a small margin (body: 0.58% for SECT, 0.72% for DECT, 0.57% for PCCT, head: 0.58% for SECT, 0.48% for DECT, 0.46% for PCCT). However, for each tissue subgroup separately, MAEs for PCCT were not consistently lowest and MAEs were comparable across imaging modalities, ranging from 0.22% to 2.83%. In the clinical cases, dose distributions for SECT‐HLUT and PCCT plans showed dose differences particularly at the distal end of the beams, attributed to optimization with reduced range uncertainty in the PCCT plan or due to areas with thicker bone or air cavities.

**Conclusions:**

The DirectSPR application for PCCT achieved promising accuracy in SPR prediction. Overall, the absolute deviations were small and comparable across all three imaging modalities. However, concerning the performance of SECT, it should be noted that the SECT‐based HLUT was calibrated using the same CIRS phantom as used during the evaluation, whereby these results are the best‐case scenario. Although the clinical cases showed minimal differences in dose distributions between SECT and PCCT plans, PCCT may offer improved reliability, as it provides patient‐specific direct SPR predictions without relying on HLUT conversion.

## 1 INTRODUCTION

A substantial source of error in proton therapy planning arises from the uncertainty in predicting the stopping power ratio (SPR) of tissues from computed tomography (CT). Currently, conventional single‐energy CT (SECT) scans are mostly used in clinical practice with a Hounsfield look‐up table (HLUT) for converting patient CT data into SPR, enabling calculations of dose depositions in treatment planning systems. Calibration of an HLUT involves correlating CT numbers (expressed in Hounsfield units, HU) to SPRs using a heuristic linear conversion approach based on phantoms with known tissue‐equivalent materials and/or tabulated tissues.^1^, ^2^ An HLUT needs to be created for each CT scanner and could be specified separately for body and head scanning protocol. Once defined, the appropriate HLUT is then applied consistently across all patients scanned with that particular scanner and protocol. A key limitation of the HLUT is its fixed CT‐number‐to‐SPR conversion, which cannot fully account for beam‐hardening effects or the variability in CT number and SPR, as tissues with the same CT number at a given tube voltage can have different SPRs, and vice versa. This is due to the fact that although both CT number and SPR are related to relative electron density (RED), the CT number also depends on the elemental composition of the tissue often described by the effective atomic number (EAN), while SPR on the other hand is impacted by the mean excitation energy (*I*).^3^ As a result, most current clinical practices with HLUT‐based methods apply a range uncertainty of 3%–3.5% in robust proton treatment planning to ensure adequate dose delivery to the tumor, which also impacts surrounding healthy tissue.^4^, ^5^, ^6^, ^7^, ^8^ Due to its systematic nature, it is crucial to minimize this error as much as possible.

To address this limitation, energy‐resolved CT scans can be used to extract more detailed information per voxel, such as RED and EAN.^9^ This enhanced data allows for a patient‐specific approach with more accurate SPR estimations.^9^, ^10^, ^11^ Various algorithms for direct SPR prediction from spectral information have been described for dual‐energy CT (DECT).^11^ However, although the use of DECT has already demonstrated a reduction in range uncertainty to 2%, this method is currently implemented in only a few clinics.^12^, ^13^, ^14^, ^15^

Beyond DECT, photon‐counting CT (PCCT) scanners, which utilize semiconductor detectors, have the potential to further enhance material characterization.^16^, ^17^ PCCT can offer improved spatial resolution, a higher signal‐to‐noise ratio, while reducing radiation doses.^18^, ^19^ These advancements could improve the accuracy of SPR prediction, which is crucial for reducing range uncertainty and enhancing proton therapy planning. Additionally, they could improve diagnosis and staging.^20^, ^21^, ^22^, ^23^

PCCT has not yet been introduced routinely in proton therapy. While theoretical simulations and experimental measurements using in‐house algorithms have suggested that PCCT may offer improved or at least comparable SPR predictions to DECT,^24^, ^25^, ^26^ these findings are still untested in a clinical setting. For this study, Siemens Healthineers provided a research software application that generates patient‐specific SPR datasets directly from PCCT, similar to the clinically available DirectSPR application for DECT.^12^, ^15^, ^27^, ^28^ Importantly, the DirectSPR application eliminates the need for HLUT specification; however, this approach has yet to be validated for PCCT. Therefore, we aim to evaluate this research DirectSPR application for PCCT and assess its accuracy in SPR prediction. The research primarily focused on technical phantom measurements, which were used to compare SPR predictions from PCCT, SECT, and DECT. In addition, a clinical evaluation was included as a proof of concept to demonstrate the potential applicability of the method.

## 2 MATERIAL AND METHODS

### 2.1 Phantom

**FIGURE 1:**
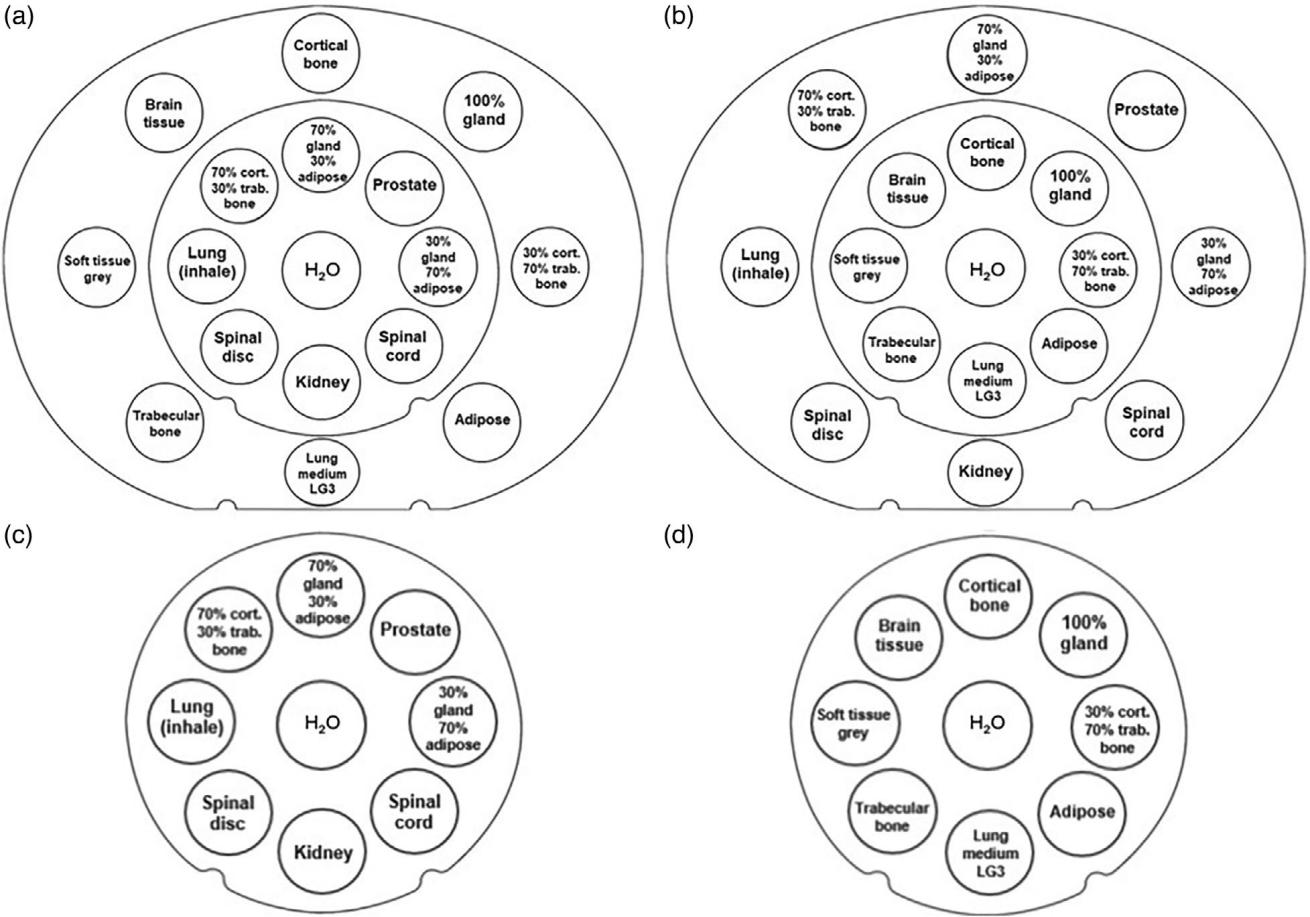
Configurations of inserts which represent different tissue surrogates in the phantom in the body (a and b) and in the head (c and d) configurations, taken from Peters et al.^6^ ^.^

The electron density CIRS phantom (Model FER 1976‐0001 062 SPR/Density Phantom), made of solid water material with tissue‐equivalent inserts in a body and head configuration, was used for the investigation. The inserts were sub‐divided into four tissue groups: lung tissues (*n* = 2), adipose‐like tissues (*n* = 4), soft tissues (*n* = 5) and bone (*n* = 5).^2^ All inserts were cylinders with a diameter of 3 and 5 cm in length. High‐ and low‐density inserts were strategically distributed throughout the phantom to minimize interference between the tissue surrogates.^6^ Two distinct body configurations were used, to place each insert once within the inner and once within the outer phantom ring (Figure 1a, b). Two head configurations were also used, to encompass all 16 inserts (Figure 1c, d).

### 2.2 Imaging

All four phantom configurations (two body and two head) were scanned in three different modes: (i) SECT and (ii) DECT at HollandPTC (Delft, the Netherlands) as well as (iii) PCCT at Erasmus MC (Rotterdam, The Netherlands). CT scanner details and the imaging acquisition parameters are listed in Table 1. For SECT and DECT, the imaging protocols for both body and head were selected CT protocols used clinically for radiotherapy. As there were no radiotherapy imaging protocols available at the PCCT scanner, the scanning parameters and reconstruction kernel for PCCT were chosen to closely replicate the imaging protocols used for SECT and DECT. For comparison reasons, the volume CT dose index (CTDIvol) was set to similar levels across all modalities (CTDIvol, 32 cm of 20 mGy for the body phantom, and CTDIvol, 16 cm of 40 mGy for the head phantom). The effect of reducing CTDIvol, 32 cm in PCCT scans on SPR predictions was investigated in both body configurations, by reducing CTDIvol, 32 cm to 10 mGy and 5 mGy.

**TABLE 1:** Scanner and imaging acquisition details for single‐energy CT (SECT), dual‐energy CT (DECT) and photon‐counting CT (PCCT).

	SECT	DECT	PCCT
CT manufacturer	Siemens Healthineers	Siemens Healthineers	Siemens Healthineers
CT scanner	SOMATOM Definition Edge	SOMATOM Definition Edge	NAEOTOM Alpha
Version	Syngo.CT VB20	Syngo.CT VB20	Syngo.CT VB10
Tube Voltage	120 kV	80/140 kV	120 kV
Scan Mode	Spiral	Dual Spiral	Spiral
CTDI (32 cm/16 cm)	20/40 mGy	20/40 mGy	20/40 mGy
Field‐of‐view (body/head)	500 mm/250 mm	500 mm/250 mm	500 mm/250 mm
Rotation time	0.5 s	0.5 s	0.5 s
Pitch	0.8	0.6/1.2	0.8 (body)/ 0.35 (head)
Detector collimation	Physical 64 x 0.6 mm Acq mode 32 x 1.2 mm	Physical 64 x 0.6 mm Acq mode 128 x 0.6 mm	Acq mode 144 x 0.4 mm (body) Acq mode 96 x 0.4 mm (head)
Slice thickness	2 mm	2 mm	2 mm
Slice increment	2 mm	2 mm	1.5 mm
Reconstruction Kernel	Qr40, SAFIRE 3, iBHC Bone	Qr40, SAFIRE 3, iBHC Bone	Qr40, QIR 3, iBHC Quantum

### 2.3 SPR determination

The SPR of each insert was determined in five different ways:

SPR_Measured_: At the clinical gantry room in HollandPTC, the water‐equivalent thickness (WET) of each insert was measured as the difference between proton range (R) with and without the insert in the beam path, with the range given as the distal 80% dose fall‐off. Measurements were performed using a multi‐layer ionization chamber (Giraffe, IBA Dosimetry, Schwarzenbruck, Germany) which has a measurement uncertainty of approximately 0.1 mm. As recommended by the consensus guide^2^ a high proton energy beam of 200 MeV was used, in order to minimize lateral beam scattering and achieve a sharper Bragg peak. The SPR of each insert was determined as follows, with *L* describing the length of each insert (50 mm):

$$\mathrm{SP}R_{\mathrm{Measured}}=\frac{\;\mathrm{WE}T_{\mathrm{insert}}}{L_{\mathrm{insert}}}=\frac{R_{\mathrm{without}\;\mathrm{insert}\;}-\;R_{\mathrm{with}\;\mathrm{insert}\;}}{L_{\mathrm{insert}}}$$

This measured SPR of each insert was considered as the ground truth.

SPR_Theoretical_: In order to validate SPR_Measured_, the SPR was determined theoretically based on the elemental composition and batch‐specific mass density information provided by the phantom manufacturer. For the theoretical SPR calculation, we used the Bethe equation^29^ and for calculating the mean excitation energy (*I)*, we used the Bragg additivity rule, where the mean excitation energies of elements where taken from ICRU report 49.^3^ A beam energy of 200 MeV was applied in the calculation, to be consistent with the beam energy used in the proton beam measurements. In addition, to align with the beam energy for the calibration of DirectSPR, we also calculated SPR_Theoretical_ at 100 MeV.

SPR_SECT_: In each SECT image, a volume of interest (VOI), covering the central volume of each insert, was delineated, excluding voxels at borders (diameter 2 cm, volume 6.25 cm^3^). The SPR was then determined within this volume, by averaging the CT numbers and applying the clinically available HLUT, which was calibrated with the CIRS phantom following the consensus guide on CT‐based prediction of SPR.^2^ As stated in the guide, theoretical SPR values at 100 MeV and measurement values at 200 MeV can be combined for HLUT specification.

SPR_DECT_: For DECT scans, the commercially available DirectSPR from Siemens Healthineers was used, which includes a size‐dependent calibration of the low‐ (80 kV) and high‐energy (140 kV) input images to directly generate an SPR dataset.^12^ Within DirectSPR, the SPR is determined as product of electron density and stopping number and the stopping number is predicted from the effective atomic number. Similar to SPR_SECT_, VOIs (diameter 2 cm, volume 6.25 cm^3^) were created on the SPR image to determine the SPR in each insert, by averaging the SPR in this volume.

SPR_PCCT_: For PCCT data, the research DirectSPR application (provided by Siemens Healthineers) was used to directly generate an SPR dataset from spectral post‐processing (SPP) images, which contain the energy‐resolved information from PCCT for spectral image post‐processing. Similar to DECT, the SPR is determined as product of electron density and stopping number, but for PCCT the stopping number is predicted from a virtual monoenergetic image.^30^ Again, identical VOIs were created on these SPR datasets to determine SPR_PCCT_ in each insert.

The DirectSPR application for DECT and PCCT is calibrated using a proton energy of 100 MeV, using custom‐made inserts and a custom‐made water‐equivalent cylindrical phantom with varying sizes ranging from 100 to 450 mm.

For the body setup, average SPR was calculated for each insert including the SPR value (SPR_SECT_, SPR_DECT_, SPR_PCCT_) of the insert estimated in the two different body configurations (Figure 1a, b). For each insert, SPR_Theoretical_ and estimated SPR_SECT_, SPR_DECT_, SPR_PCCT_ were compared to the ground truth SPR_Measured_, and SPR differences relative to water (SPR = 1) were calculated:

$$\Delta \mathrm{SPR}=\frac{\mathrm{SP}R_{\mathrm{Theorethical},\mathrm{SECT},\mathrm{DECT},\mathrm{PCCT}}-\;\mathrm{SP}R_{\mathrm{Measured}\;}}{\mathrm{SP}R_{\mathrm{Water}}}\times 100\%$$

Mean absolute errors (MAE) were calculated over SPR for all inserts in both body and head configurations and per tissue subgroup:

$$\mathrm{MAE}=\frac{1}{n}\;\sum_{i=\mathrm{insert}}^{n}\left|\;\Delta \mathrm{SP}R_{i}\right|$$

### 2.4 Proof‐of‐concept for two clinical cases

As a first proof‐of‐concept including a clinical evaluation, two patient cases were assessed to compare the dose differences between using the SECT‐HLUT or DirectSPR for PCCT, while using reduced range uncertainty settings for the PCCT plan. We retrospectively identified patients scanned at the PCCT scanner at the Erasmus MC who also received photon radiotherapy within one month. We selected one lung cancer case and one neurological case to evaluate. Inclusion also required the availability of an SPP dataset in order to reconstruct a virtual monoenergetic image (VMI) of 70 keV in syngo.via (version VB80c) to mimic a 120 kV SECT image.^27^ This procedure was verified by comparing CT numbers in the 70 keV VMI reconstructed from the SPP dataset of PCCT and 120 kV image from SECT scanner of the CIRS phantom. Differences in CT numbers ranging from 0.2 HU up to 113 HU (cortical bone) were accepted.

For the lung case, the clinical target volume (CTV) from the clinical photon plan was representative for a proton case, thus this CTV was deformably registered to the 70 keV VMI dataset and used in the creation of the proton treatment plan. For the neurological case, a hypothetical brain target volume, located in the posterior left region of the brain tissue, was manually delineated on the 70 keV VMI dataset. Two proton plans for each case were robustly optimized in Raystation (version 2023B). One plan was created on the VMI at 70 keV, using the clinical HLUT, and range and setup uncertainties of 3% and 3 mm, respectively, were applied in the robust optimization. This plan was recalculated on the PCCT‐derived SPR dataset to enable direct dose comparison with the second plan which was created on this PCCT‐derived SPR dataset, and robustly optimized using reduced range uncertainty settings of 2% (as shown feasible^12^), while keeping the setup uncertainty at 3 mm. Dose distributions on the SPR dataset were compared and dose differences were analyzed visually. For the neurological case (planned with two beams), as plans were created using single‐field uniform dose, single field dose distributions were compared, and absolute deviations in beam range (in mm) were evaluated using line‐dose profiles along the central beam axis. The lung case was planned using three beams and multi‐field optimization, with the complete plans compared and dose differences analyzed. Since this was a proof of concept evaluation, motion management strategies, repainting, and robust evaluation methods were not considered.

## 3 RESULTS

**FIGURE 2:**
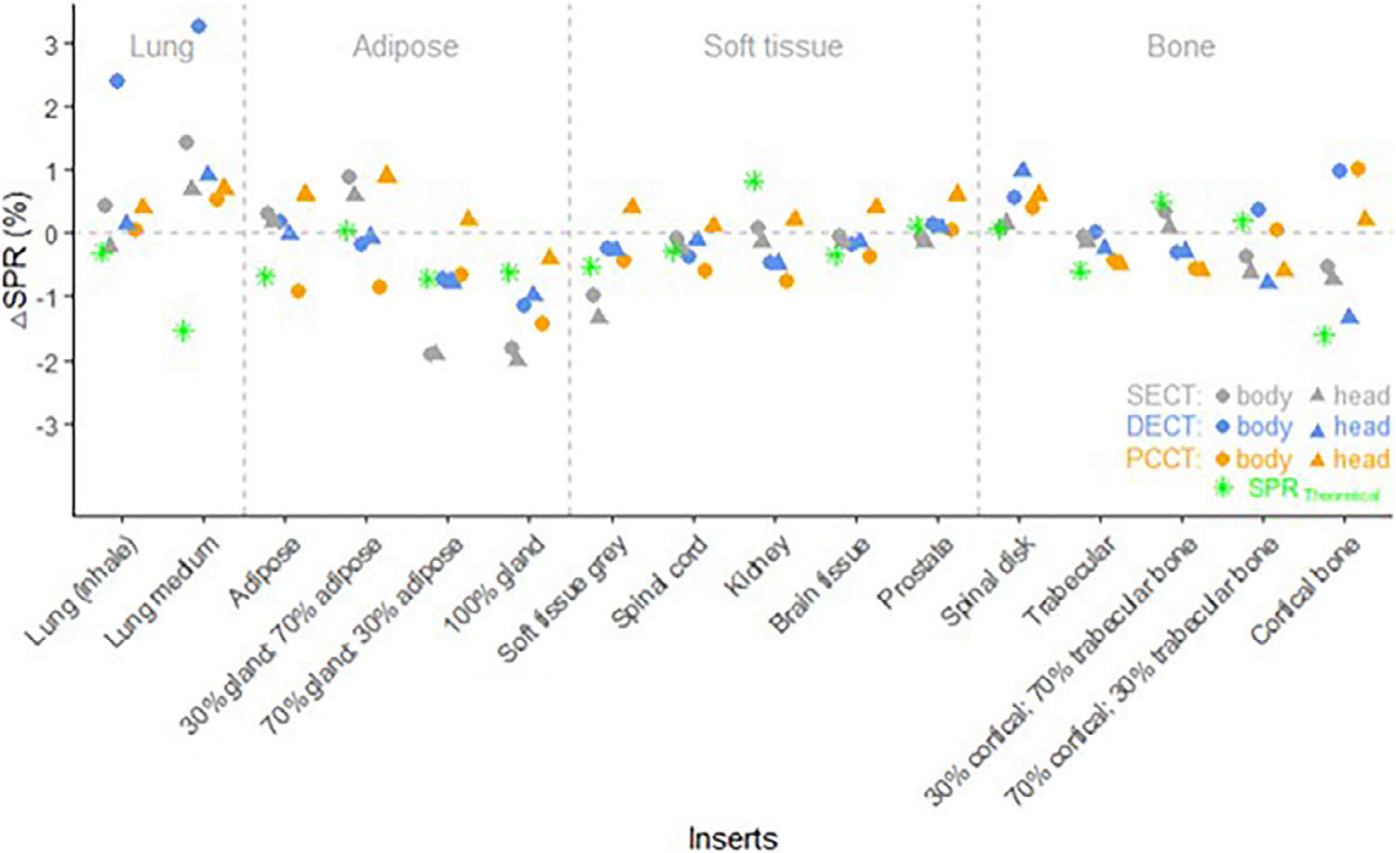
Deviations in stopping‐power ratio prediction (∆SPR) with respect to SPR_Measured_ and relative to water for individual inserts in the CIRS body (circles) and head (triangles) phantom configurations calculated using the SECT‐HLUT approach (gray), and DirectSPR with DECT (blue) and PCCT (orange). Green stars indicate differences between SPR_Theoretical_ (at 200 Mev) and SPR_Measured_.

SPRs for each insert are listed in Table 2. Differences between SPR_Theoretical_ and SPR_Measured_ were ≤1.0% (shown as green crosses in Figure 2), except of lung medium (1.5%) and cortical bone (1.6%) inserts. Differences in SPR estimation for each insert between the two body setups were less than 0.7%, except for the cortical bone insert (1.3%). PCCT predicted SPR within 1.0% agreement with SPR_Measured_, except for the insert “100% gland” (1.4%). Overall, the largest SPR deviations were found in lung tissue surrogates and adipose‐like tissues (Figure 2). For soft tissue and bone inserts, all estimated SPRs generally agreed well with SPR_Measured_ (∆SPR < 1.0%), except for the inserts soft tissue grey and cortical bone in the head configuration (both ∆SPR_SECT_ = 1.3%).

**TABLE 2:** Stopping power ratio (SPR) of each insert derived by range measurements (SPR_Meas_), theoretically calculated (SPR_Theor_, at 200 and 100 MeV) and predicted by single‐energy CT (SECT), dual‐energy CT (DECT) and photon‐counting CT (PCCT), sub‐divided into four tissue groups indicated with dashed lines. The inserts were CT scanned in body and head phantom configurations. For the body setup, average SPR was calculated for each insert including the SPR value (SPR_SECT_, SPR_DECT_, SPR_PCCT_) of the insert estimated in the two different body configurations.

					Body			Head		
Inserts	Density (g/cm^3^)	SPR_Meas_ _200 MeV_	SPR_Theor_ _200 MeV_	SPR_Theor_ _100 MeV_	SPR_SECT_	SPR_DECT_	SPR_PCCT_	SPR_SECT_	SPR_DECT_	SPR_PCCT_
Lung (inhale)	0.206	0.206	0.203	0.203	0.210	0.230	0.206	0.204	0.207	0.210
Lung medium	0.306	0.312	0.297	0.297	0.326	0.345	0.317	0.319	0.321	0.319
Adipose	0.958	0.970	0.963	0.964	0.973	0.972	0.961	0.972	0.970	0.976
30% gland; 70% adipose	0.974	0.974	0.974	0.976	0.983	0.972	0.965	0.980	0.973	0.983
70% gland; 30% adipose	1.013	1.018	1.011	1.012	0.999	1.011	1.012	0.999	1.010	1.020
100% gland	1.046	1.048	1.042	1.043	1.030	1.037	1.034	1.028	1.038	1.044
Soft tissue gray	1.056	1.038	1.033	1.033	1.028	1.036	1.034	1.025	1.035	1.042
Spinal cord	1.058	1.036	1.033	1.033	1.035	1.032	1.030	1.034	1.035	1.037
Kidney	1.068	1.040	1.048	1.049	1.041	1.035	1.032	1.038	1.035	1.042
Brain tissue	1.069	1.042	1.039	1.039	1.042	1.040	1.038	1.041	1.041	1.046
Prostate	1.079	1.040	1.041	1.041	1.040	1.041	1.041	1.039	1.041	1.046
Spinal disk	1.128	1.070	1.071	1.070	1.071	1.076	1.074	1.072	1.080	1.076
Trabecular bone	1.196	1.156	1.150	1.150	1.156	1.156	1.151	1.155	1.154	1.151
30% cortical; 70% trabecular bone	1.340	1.258	1.263	1.262	1.261	1.255	1.252	1.259	1.255	1.252
70% cortical; 30% trabecular bone	1.615	1.470	1.472	1.468	1.466	1.474	1.471	1.464	1.462	1.464
Cortical bone	1.890	1.686	1.670	1.664	1.681	1.696	1.696	1.679	1.673	1.688

Among the three imaging modalities, when comparing SPR prediction from PCCT to SECT and DECT, the overall mean absolute error (MAE) was lowest for PCCT by a small margin (body: 0.58% for SECT, 0.72% for DECT, 0.57% for PCCT, head: 0.58% for SECT, 0.48% for DECT, 0.46% for PCCT). However, for each tissue subgroup, MAEs for PCCT were not consistently lowest and MAEs across the three imaging modalities were small and comparable, ranging from 0.22% to 2.83% (Table 3).

**TABLE 3:** Mean absolute error (MAE in %) of stopping power ratio (SPR) between range measurements (SPR_Measured_) and single‐energy CT (SECT), dual‐energy CT (DECT) and photon‐counting CT (PCCT) over all inserts and per tissue‐subgroup.

	Body			Head		
	SECT	DECT	PCCT	SECT	DECT	PCCT
All inserts	0.58	0.72	0.57	0.58	0.48	0.46
Lung	0.93	2.83	0.29	0.45	0.52	0.55
Adipose	1.23	0.56	0.97	1.18	0.46	0.52
Soft tissue	0.24	0.28	0.45	0.40	0.22	0.34
Bone	0.27	0.44	0.49	0.35	0.73	0.50

The effect of reducing CTDIvol in PCCT scans on SPR predictions showed absolute differences ≤ 0.006 (relative ≤ 0.63%) per insert between SPR predictions in PCCT scans with CTDIvol ranging from 20 mGy to 5 mGy.

**FIGURE 3:**
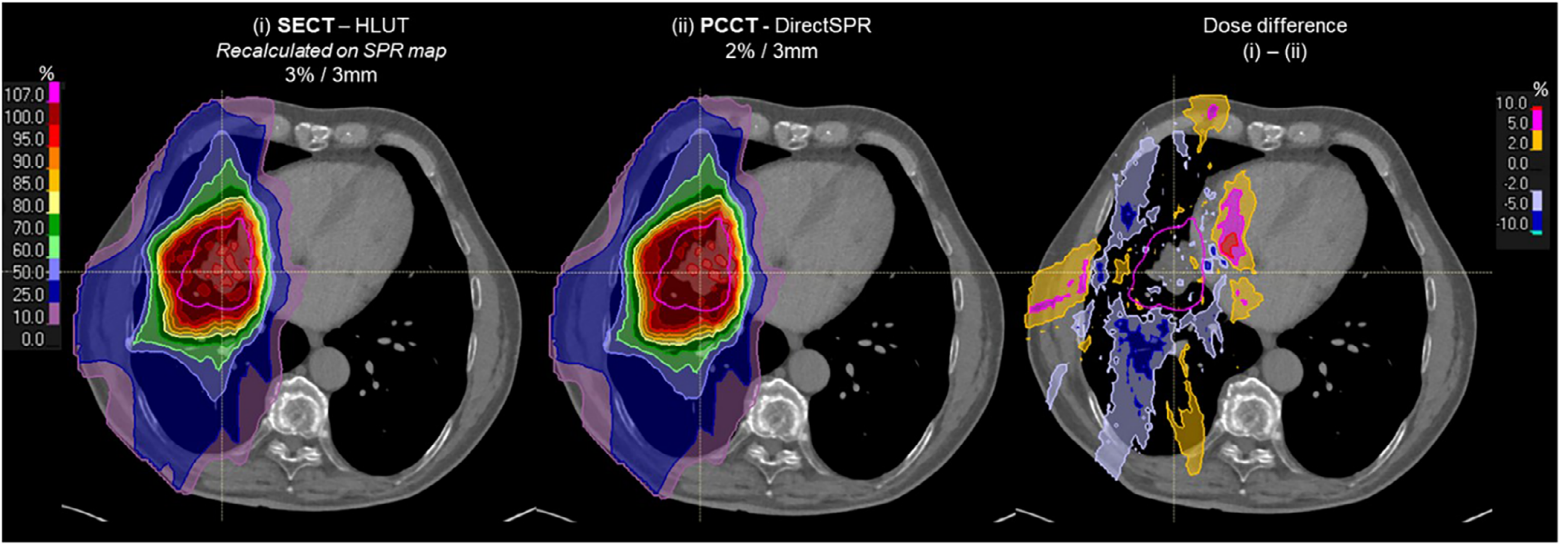
A three‐beam proton therapy plan was created, with beams directed from the posterior (200°), anterior (345°), and lateral (250°) angles to optimize tumor (delineated in magenta) coverage and minimize exposure to surrounding tissues. Dose distributions for a lung patient (i) optimized on the 70 keV VMI dataset using the HLUT approach with 3%/3 mm uncertainty settings and recalculated on the PCCT‐derived SPR dataset and (ii) optimized with 2% range uncertainty on the SPR image from the research DirectSPR application for PCCT, as well as the dose difference (SECT‐plan minus PCCT‐plan). The colorbar on the left indicates the relative dose with respect to the prescription dose.

**FIGURE 4:**
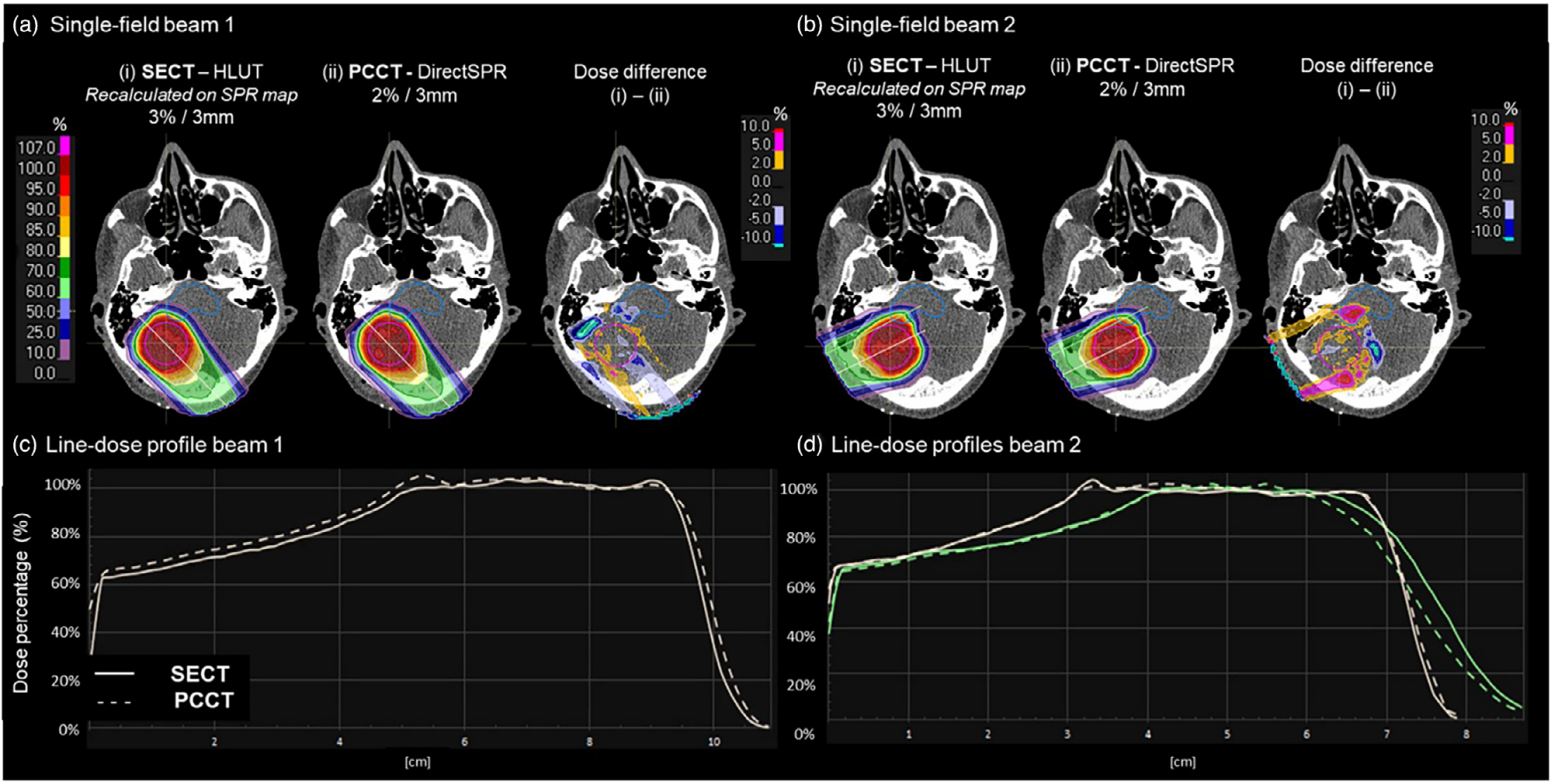
(a‐b) Single‐field dose distributions for a neurological patient (i) optimized on the 70 keV VMI dataset using the HLUT approach with 3%/3 mm uncertainty settings and recalculated on the PCCT‐derived SPR dataset and (ii) optimized with 2% range uncertainty on the SPR image from the research DirectSPR application for PCCT, as well as the dose difference (SECT‐plan minus PCCT‐plan). The hypothetical brain target volume (magenta) and the brainstem (blue) are delineated. The colorbar on the left indicates the relative dose with respect to the prescription dose per beam. (c‐d) Line‐dose profiles, corresponding to the line profiles along the central beam axis shown in A‐B in bright color, to indicate deviations in range predictions. An additional line‐dose profile (green in B and D) was taken at the location where the beam passed through the ear cavity's air‐tissue interface.

In the clinical example cases, the observed dose differences between the SECT‐HLUT plans recalculated on the PCCT‐derived SPR dataset and the PCCT‐DirectSPR plans were influenced by the types of tissues encoutered along the beam path, as well as differences in the range uncertainty settings applied during optimization. For the lung case, no differences were observed in the target volume (Figure 3), while larger dose differences were mainly attributed to optimization with reduced range uncertainty in the PCCT plan. For organs at risk in close proximity to the target, such as the heart and lungs, minimal dose differences (< 0.5 Gy) were found between the two plans. For the neurological case, single‐field dose distribution from the first beam showed that the high‐dose region proximal to the target volume was more conformal in the SECT‐based plan compared to the PCCT plan. At this location, the beam has passed through bone and soft tissue. The resulting differences suggest that the estimated SPRs based on the SECT‐HLUT and DirectSPR PCCT approaches may have led to differences in proton range. As the beam continued through soft tissue in the target volume, this difference appeared to compensate, resulting in comparable dose distributions (Figure 4a) and similar beam profiles (Figure 4c). An exception to this can be seen when the beam passes through bony structures at the distal end, particularly near the inner ear, where a larger dose difference was noted and a range difference of 1 mm (Figure 4c). In the second beam, minimal differences were observed along the central beam line through the axis (Figure 4b). An additional dose profile was taken at the location where the beam passed through the ear cavity's air‐tissue interface, revealing the impact of differences in SPR estimation between the SECT‐HLUT and DirectSPR PCCT approach. A dose difference up to 10% and range difference of 2 mm was observed at the distal end (Figure 4d), located near/within the brainstem (outlined in blue), which could be clinically relevant.

## 4 DISCUSSION

This study highlights the promising accuracy of the DirectSPR application for PCCT in SPR prediction, achieving agreement ≤1.0% compared to SPR_Measured_, with the exception of the insert “100% gland”, which showed a deviation of 1.4%. Overall absolute deviations were small and comparably accurate between all imaging modalities. This study demonstrated that DirectSPR for PCCT can be integrated into the treatment workflow by generating an SPR dataset directly imported into the treatment planning software. Dose differences between the SECT‐HLUT and PCCT plans in clinical cases were influenced by differences in SPR estimations particularly for bone or air tissue types as well as reduced range uncertainty. While the clinical cases showed minimal differences, PCCT may offer improved reliability, as it provides patient‐specific direct SPR determination without relying on HLUT conversion.

We acknowledge that our VOI analysis for estimating SPR_SECT_ was more straightforward compared to the voxel‐wise SPR estimation used for SPR_DECT_ and SPR_PCCT_. However, since the HLUT is a piecewise linear function, we are confident that averaging the CT numbers within the volume provided a valid estimation of applying the HLUT for estimating SPR_SECT._ While SPR_SECT_ showed good performance, it should be noted that the SECT‐based HLUT was calibrated using the same CIRS phantom, following the step‐by‐step consensus guide on CT‐based prediction of SPR.^2^ In this calibration process, SPR calculations were based on proton range experiments of the same inserts and Bethe calculations of tabulated tissues, meaning that SPR_SECT_ results are a best‐case scenario, explaining the minimal deviations between SPR_SECT_ and SPR_Measured_. This calibration approach may also account for the differences between our findings and previous studies, which reported greater improvements in SPR predictions using spectral imaging compared with SECT, as summarized in review papers.^9^, ^11^

To evaluate a more realistic performance of the SECT‐HLUT approach, future studies could incorporate biological experiments including animal tissues, as previously demonstrated in DECT studies.^31^, ^32^, ^33^ When using biological tissue samples, these tissues differ from the insert materials used in the calibration process. Even if the CT numbers of the samples are the same, their SPR may differ,^34^, ^35^ a variation that would not be detected by the SECT‐HLUT approach. However, SPR derived from spectral information might be able to identify these differences, enabling more accurate SPR prediction. Another suggestion for further investigation could be using the same insert materials as those used in the calibration process, but testing with phantoms of varying sizes.

SPR_Theoretical_ is subject to potential uncertainties since the mass density and elemental composition of the phantom inserts were provided by the manufacturer and could not be independently verified. Moreover, the *I* values used in the Bethe equation, as used from the ICRU report^3^ also come with inherent uncertainties. Therefore, comparing the predicted SPR_SECT/DECT/PCCT_ to SPR_Measured_ offers a more reliable assessment. In this study, we chose to report relative differences to water, aligning with the clinical context where water is universally used as the reference medium in proton therapy for dosimetry and range calculations.

In this study, we focused on CT‐based estimation of SPR, which is a substantial contributor to the range uncertainty.^4^ However, we did not consider other clinically relevant contributions, such as imaging, modeling and reconstruction uncertainties.^12^ Peters et al. investigated the commercially available DirectSPR application for DECT in 150 brain‐ and 100 prostate‐cancer patients, and proposed a site‐specific range uncertainty of 1.7%/2 mm and 2%/2 mm^12^ respectively, closely aligning with other studies reporting values of 2.4%/1.2 mm^33^ and 2.2%.^36^ Our findings indicate that PCCT could also potentially reduce overall CT‐related range uncertainties compared to SECT‐HLUT approach. Consequently, we applied the clinical uncertainty settings of 3%/3 mm in our clinical evaluations for the SECT‐HLUT approach and reduced range uncertainty 2% for the DirectSPR application for PCCT. In line with previous literature on DECT, we observed that dose discrepancies were most prominent at the distal end of the beams.^28^, ^34^, ^37^ Reductions in range uncertainty have shown clinical benefits for neuro‐oncology patients.^14^, ^38^, ^39^ For patients with head‐and‐neck cancer, set‐up uncertainties appear to be more critical than range uncertainties.^40^, ^41^

In line with previous studies comparing SPR predictions between DECT and PCCT^24^, ^25^, ^26^ our findings also showed small differences and comparable results between the two modalities. A limitation of this study is that the phantom used was static and could not demonstrate the advantage of PCCT over DECT in terms of temporal coherence. PCCT offers superior temporal coherence compared to DECT techniques, which can be affected by time delays inherent to DECT scanning techniques, such as dual‐source, DualSpiral/TwinSpiral, TwinBeam, and kV‐switching methods. This advantage could reduce potential artifacts and enhances image consistency, making it particularly valuable for treatment areas prone to movement, such as the thoracic or abdominal regions.

Exploratory scans showed that with PCCT we could potentially reduce the radiation dose (expressed by CTDIvol) with no relevant differences in SPR predictions, which is in accordance with others who demonstrated that low‐dose CT is suitable for proton dose calculations.^42^, ^43^ Although imaging doses are not a primary concern given the relatively high treatment dose in proton therapy, this feature could be particularly beneficial for children, due to their increased sensitivity to radiation and the potential long‐term risks of exposure. The combination of lower imaging doses, higher spatial resolution for delineation and accurate SPR predictions makes PCCT a promising tool for pediatric proton therapy.

Further studies on all above‐mentioned subjects will be needed to be able to utilize the full potential of PCCT in proton therapy. While SECT provides only a single attenuation value, PCCT enables energy‐resolved imaging for material differentiation, making it more adequate for estimating SPR. Consequently, DirectSPR provides a more robust approach and is patient‐specific by eliminating the need for a generic HLUT. This is particularly important for tissues whose properties deviate from the calibration standards. Furthermore, PCCT phantom measurements demonstrated promising results for SPR predictions, contributing to accurate and reliable range predictions. Although limited to two clinical cases, these served as a proof of concept for the potential advantages of PCCT in accurate and individualized proton treatment planning.

## CONFLICT OF INTEREST STATEMENT

P. Wohlfahrt is employed by Siemens Healthineers within the research and development department. The department of Radiotherapy and the department of Radiology & Nuclear Medicine (Erasmus MC) and HollandPTC all have a research agreement with Siemens Healthineers in the field of photon‐counting CT, as well as a software evaluation contract.
